# The Psychrotrophic Pseudomonas lundensis, a Non-*aeruginosa* Pseudomonad, Has a Type III Secretion System of the Ysc Family, Which Is Transcriptionally Active at 37°C

**DOI:** 10.1128/mbio.03869-21

**Published:** 2022-02-22

**Authors:** Keerthikka Ravi, Nicole R. Falkowski, Brittan S. Scales, Volha D. Akulava, Leonid N. Valentovich, Gary B. Huffnagle

**Affiliations:** a Department of Molecular, Cellular & Developmental Biology, Ann Arbor, Michigan, USA; b Department of Microbiology & Immunology, University of Michigan, Ann Arbor, Michigan, USA; c Division of Pulmonary & Critical Care Medicine, University of Michigan, Ann Arbor, Michigan, USA; d Mary H. Weiser Food Allergy Center, University of Michigan, Ann Arbor, Michigan, USA; e Faculty of Biology, Belarusian State University, Minsk, Belarus; f Institute of Microbiology, National Academy of Sciences of Belarus, Minsk, Belarus; g Faculty of Science and Technology, Norwegian University of Life Sciences, Ås, Norway; University of Maryland, School of Medicine

**Keywords:** 2T.2.5.2, AU1044, Antarctica, M101, M105, *Pseudomonas fluorescens*, milk, spoilage

## Abstract

The type III secretion system (T3SS) is a needle-like structure found in Gram-negative pathogens that directly delivers virulence factors like toxins and effector molecules into eukaryotic cells. The T3SS is classified into different families according to the type of effector and host. Of these, the Ysc family T3SS, found in *Yersinia* species and Pseudomonas aeruginosa, confers high virulence to bacteria against eukaryotic hosts. Here, we present the first identification and transcriptional analyses of a Ysc T3SS in a non-*aeruginosa* Pseudomonas species, Pseudomonas lundensis, an environmental psychrotrophic bacterium and important agent of frozen food spoilage. We have identified and sequenced isolates of P. lundensis from three very distinct ecological niches (Antarctic temporary meltwater pond, U.S. supermarket 1% pasteurized milk, and cystic fibrosis lungs) and compared these to previously reported food spoilage isolates in Europe. In this paper, we show that strains of P. lundensis isolated from these diverse environments with ambient temperatures ranging from below freezing to 37°C all possess a Ysc family T3SS secretion system and a T3S effector, ExoU. Using *in vitro* and *in vivo* transcriptomics, we show that the T3SS in P. lundensis is transcriptionally active, is expressed more highly at mammalian body temperature (37°C) than 4°C, and has even higher expression levels when colonizing a host environment (mouse intestine). Thus, this Ysc T3SS-expressing psychrotrophic Pseudomonad has an even greater range of growth niches than previously appreciated, including diseased human airways.

## INTRODUCTION

Pseudomonas lundensis is a psychrotrophic bacterium whose extent of distribution across diverse environments has not been fully appreciated. P. lundensis was first reported in 1986 as a prime spoilage bacterium in cold beef and pork (1) and has since been found growing in chilled meat, milk, and milk products (2–4). It has also been isolated from the feces of wild boar (5), Antarctic temporary meltwater ponds, and sputum samples in patients with cystic fibrosis (6), all of which demonstrate the wide diversity of environments in which this bacterium is able to persist and grow. P. lundensis has favorable growth temperatures between 0°C to 30°C, with optimal growth at 25°C (1). We can readily culture this bacterium at 37°C, indicating that the upper temperature limit for growth of this psychrotroph is at least 37°C. P. lundensis is a Gram-negative, polar flagellated bacterium belonging to the Pseudomonas fragi clade of the Pseudomonas fluorescens species complex (7). This bacterium exhibits surface-associated motility and is capable of forming stable biofilm structures across a wide temperature range (8). However, we report in this paper that P. lundensis also encodes a Ysc family type III secretion system (T3SS), similar to that seen in P. aeruginosa and *Yersinia* spp.

T3SS is a molecular system that mediates cell-to-cell interactions between Gram-negative bacteria and eukaryotes. There are several families of T3SS, classified based on phylogenetic analysis, all varying in their effector and the hosts with which they interact (9). Depending on the bacterial species and host, the T3SS can sway the interaction outcomes from commensalism to symbiotic or mutualistic (e.g., Rsp family T3SS in P. fluorescens [10, 11]) or pathogenic (e.g, Ysc family, Inv-Mxi Spa family, and Hrc Hrp1/2 family T3SS in Gram-negative pathogens like P. aeruginosa, Salmonella enterica serovar Typhimurium, and Pseudomonas putida [9, 12, 13]).

In P. aeruginosa, the Ysc (Psc) family T3SS is one of many virulence characteristics in its arsenal. Although it is not essential for causing infection, the T3SS plays a very important role in interacting with a host and establishing chronic infections by dampening host defense and activating inflammasome signaling (14, 15). P. aeruginosa encodes up to four T3SS effector proteins that have known toxic activities (ExoS, ExoT, ExoY, and ExoU) of which ExoU-mediated cytotoxicity is the most potent (16, 17). However, these potent virulence characteristics are not unique to clinical isolates and can also be found in environmental isolates of P. aeruginosa (17), where T3SS confers a fitness advantage to the bacterium (18). To date, P. aeruginosa is the only Pseudomonas species in which a functional Ysc T3SS and its effector proteins have been reported.

In this current study, we have cultured, identified, and sequenced isolates of P. lundensis grown from three very distinct ecological niches (Antarctic temporary meltwater pond, U.S. supermarket pasteurized milk, and human cystic fibrosis lungs) and compared these to previously reported food spoilage isolates from Europe. The isolates described here all grow in very different environments in terms of nutritional availability, temperature, and environmental pressures, Yet strains with a complete genome assembly share >99% sequence similarity (draft genomes share >98% sequence identity). We report the identification of a complete Ysc T3SS complex in P. lundensis, as well as homologs of the ExoU effector protein and its chaperone SpcU. Like P. aeruginosa, all isolates of P. lundensis (environmental and clinical) encode a complete Ysc T3SS. The clinical isolate AU1044 expressed its T3SS in *in vitro* growth conditions at 37°C while having no expression at low temperature, 4°C. High T3SS expression is also seen in bacteria colonizing the intestinal tract of gnotobiotic mice, raising further questions about how this bacterium may interact with a mammalian host.

## RESULTS

### Isolation and characterization of P. lundensis strains from diverse environments.

Pseudomonas lundensis, commonly reported as a psychrotrophic food spoilage bacterium (for example, in cold storage spoilage of pasteurized milk), can colonize the lungs of cystic fibrosis (CF) patients (6). Strain AU1044, the strain studied in this paper, is one such clinical isolate of P. lundensis and was isolated from a sputum sample of a CF patient in Columbia, Missouri, United States (6). To test if this clinical isolate was able to grow in the known environmental niches of P. lundensis, AU1044 was cultured in milk and LB broth at ambient and cold temperatures, representing environments similar to that of spoiled foods. Under aerobic conditions, strain AU1044 was successfully grown in both milk media and LB broth and at temperatures ranging from 4°C to 37°C (data not shown). The generation times at room temperature through 37°C were similar, while the generation time at 4°C was significantly longer. Thus, the clinical isolate AU1044 can grow in cold storage food spoilage conditions similar to what has been reported for other isolates of P. lundensis (19). This was also true for the 11 other clinical isolates (AU11122, AU11136, AU11164, AU11235, AU7350, AU12597, AU12644, AU2390, AU10414, AU9518, and AU14641) that we reported earlier, which were isolated from cystic fibrosis patients from seven other sites across the United States (Ann Arbor, Michigan; Boston, Massachusetts; Chapel Hill, North Carolina; Cincinnati, Ohio; Hartford, Connecticut; Palo Alto, California, and St. Louis, Missouri) (6). Strain 2T.2.5.2 was isolated during the 5th Belarussian Antarctic Expedition (58th Russian Antarctic Expedition) in the area of the base Gora Vechernyaya from the temporary meltwater ponds (puddles) of the Lazurnaya Bay, Alasheev Bay, and Cosmonauts Sea. Strains M101 and M105 were isolated from 1% milkfat pasteurized milk, purchased from a grocery store in Ann Arbor, Michigan, in which the milk had been allowed to spoil after extended storage at 4°C. Strains 2T.2.5.2, M101, and M105 were all successfully cultured in milk media and LB broth and at temperatures ranging from 4°C to 37°C. Thus, the isolates from these extremely diverse environments (human lungs, spoiled milk, and Antarctic temporary meltwater ponds) all behaved similarly under a range of *in vitro* conditions.

### Genetic organization of P. lundensis T3SS.

**FIG 1:**
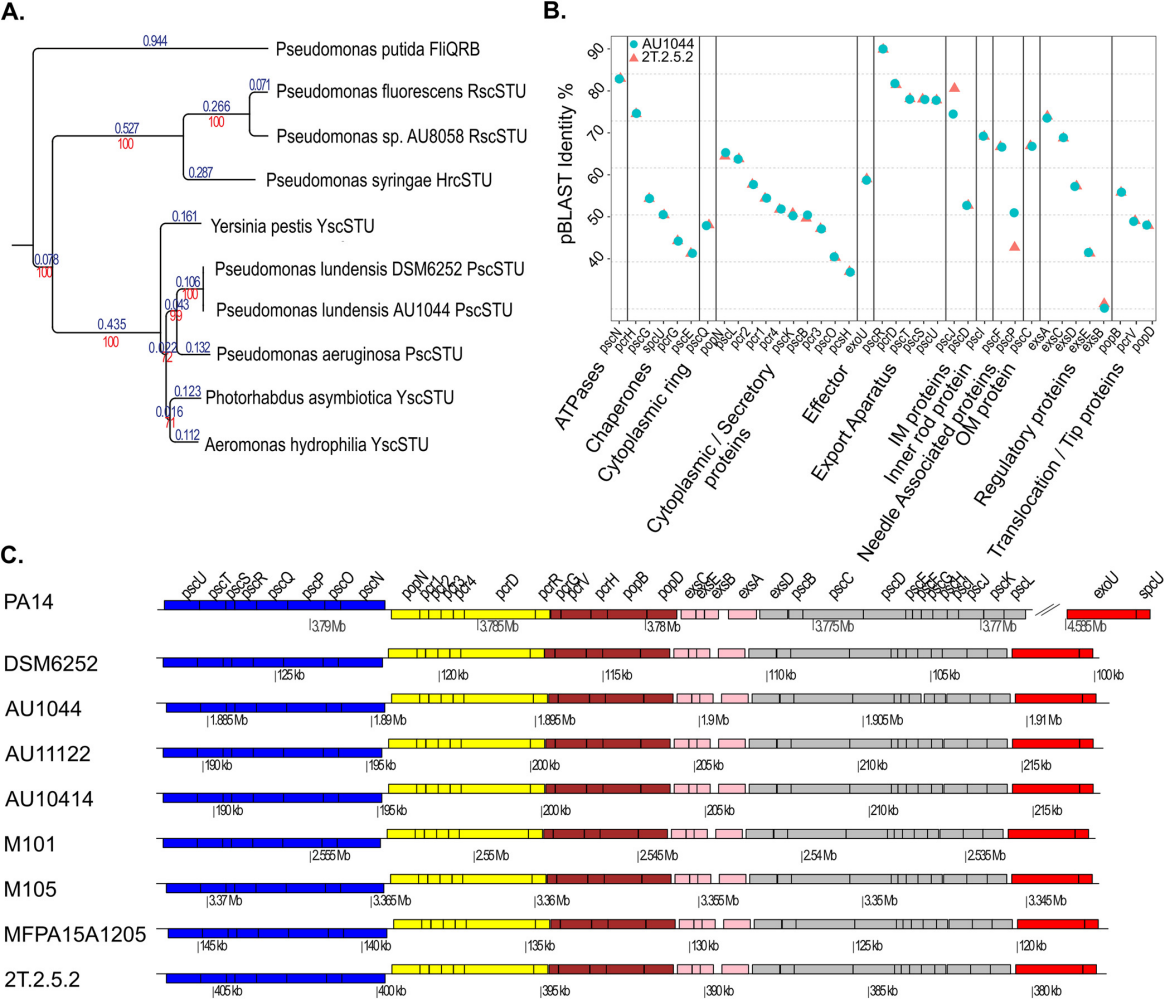
Ysc family T3SS in P. lundensis. (A) The T3SS of P. lundensis is most homologous to that of P. aeruginosa. Phylogenetic tree depicting the similarity between T3SS among Pseudomonas species discussed in this paper. The tree was built on the predicted amino acid sequences of the T3SS structural proteins PscS/Ysc, PscT/YscT, PscU/PscT, and their T3SS homologues in the other Pseudomonas species. Branch length is on the top side of branch and labeled in blue; confidence intervals based on 1,000 bootstraps of the tree are on the underside of the branch and are labeled in red. (B) pBLAST of T3SS protein from an environmental and clinical strain of P. lundensis, 2T.2.5.2 and AU1044, respectively. The BLAST analysis was done against the Psc family protein from P. aeruginosa strain PA14. (C) Gene maps of the type III secretion system from P. aeruginosa strain PA14 and P. lundensis strains DSM6252, AU104, AU11122, AU10414, M101, M105, MFPA15A1205, and 2T.2.5.2. DSM6252 is the type strain isolated from spoiled beef. AU1044, AU11122, and AU10414 are clinical isolates of P. lundensis from sputum samples of patients with cystic fibrosis. M101 and M105 are strains isolated from spoiled milk, MFPA15A1205 is a strain from a spoiled food source, and 2T.2.5.2 was isolated from an Antarctic temporary meltwater pond. In PA14, the exoU and spcU are not present immediately downstream to the core genes as seen in P. lundensis. The gene annotations seen for PA14 also apply to the P. lundensis strains.

We screened the genome of strain AU1044 using the Virulence Factors of Pathogenic Bacteria (VFDB; http://www.mgc.ac.cn/VFs/) database as a reference and identified a set of genes that were identified as Ysc family T3SS genes. A phylogenetic analysis of T3SS structural proteins PscS, PscT, and PscU from P. lundensis strains and their respective homologs showed that the T3SS in P. lundensis is most homologous to that of P. aeruginosa and falls among other species that carry Ysc family T3SS (Fig. 1A). A protein BLAST analysis was performed with the putative T3SS genes identified in AU1044 and 2.T.2.5.2 (Fig. 1B; Table 1), setting the Psc family T3SS in P. aeruginosa PA14 as the reference. Results from this analysis further confirmed that P. lundensis has a Ysc family T3SS, and this system shares high protein sequence similarities with the Psc family T3SS in P. aeruginosa.

**TABLE 1:** P. lundensis T3SS genes BLASTp analysis against Psc family T3SS in P. aeruginosa

Functional name	Sct name^*a*^	Psc name^*b*^	Data for:				Alignment score (%)	Query cover (%)	E value
			P. aeruginosa PA14		P. lundensis AU1044				
			UniProtKB ID	Protein sequence length (aa)	GenPept accession no.	Protein sequence length (aa)			
Export apparatus switch protein	sctU	pscU	Q02KI1_PSEAB	349	AOZ12679	348	79.472	97	1.04E-177
Minor export apparatus protein	sctT	pscT	Q02KI2_PSEAB	262	AOZ12680	262	79.615	97	4.80E-127
Minor export apparatus protein	sctS	pscS	Q02KI3_PSEAB	88	AOZ15077	88	79.545	99	4.53E-37
Minor export apparatus protein	sctR	pscR	Q02KI4_PSEAB	217	AOZ12681	219	90.278	100	2.49E-137
C-ring protein	sctQ	pscQ	Q02KI5_PSEAB	309	AOZ12682	308	52.787	98	4.96E-85
Needle length determinant protein	sctP	pscP	WP_010895580 (GenPept)	369	AOZ15078	403	55.385	98	2.32E-23
Stalk	sctO	pscO	Q02KI7_PSEAB	158	AOZ12683	155	46	76	1.47E-31
ATPase	sctN	pscN	Q02KI8_PSEAB	440	AOZ15079	440	84.018	99	<1E-200
Gatekeeper	sctW	popN	Q02KI9_PSEAB	288	AOZ12684	219	68.198	97	6.53E-139
		pcr1	Q02KJ0_PSEAB	92	AOZ12685	91	58.621	95	2.69E-38
		pcr2	Q02KJ1_PSEAB	123	AOZ12686	123	61.475	99	1.35E-52
		pcr3	Q02KJ2_PSEAB	121	AOZ12687	121	52.066	100	8.14E-36
		pcr4	Q02KJ3_PSEAB	109	AOZ12688	110	56.19	84	1.52E-33
Major export apparatus protein	sctV	pcrD	Q02KJ4_PSEAB	706	AOZ12689	707	83.027	100	<1E-200
		pcrR	Q02KJ5_PSEAB	144	prot_1660	144	60.67	61	1E-34
PcrV chaperone protein		pcrG	Q02KJ6_PSEAB	98	AOZ12690	94	49.425	92	3.09E-17
Tip protein		pcrV	Q02KJ7_PSEAB	294	AOZ12691	314	53.676	86	1.46E-85
Translocon chaperone protein		pcrH	Q02KJ8_PSEAB	164	AOZ12692	161	76.582	95	1.85E-84
Translocon protein		popB	Q02KJ9_PSEAB	390	AOZ12693	388	59.74	98	5.77E-141
Translocon protein		popD	Q02KK0_PSEAB	295	AOZ15080	295	52.881	100	5.69E-101
Regulatory protein		exsC	Q02KK1_PSEAB	145	AOZ12694	147	71.429	100	9.63E-65
Regulatory protein		exsE	Q02KK2_PSEAB	81	AOZ12695	81	46.914	97	1.62E-18
Regulatory protein		exsB	Q02KK3_PSEAB	137	AOZ12696	134	35.246	88	3.37E-20
Regulatory protein		exsA	Q02KK4_PSEAB	278	AOZ12697	270	75.646	100	1.40E-157
Regulatory protein		exsD	Q02KK5_PSEAB	276	AOZ12698	277	61.091	99	1.68E-121
		pscB	Q02KK6_PSEAB	140	AOZ12699	140	54.93	100	4.09E-46
Secretin	sctC	pscC	Q02KK7_PSEAB	600	AOZ15081	597	69.631	95	<1E-200
Outer MS ring protein	sctD	pscD	Q02KK8_PSEAB	432	AOZ12700	422	57.109	99	6.31E-169
PscF-stabilizing protein		pscE	Q02KK9_PSEAB	67	AOZ12701	68	46.875	94	8.30E-15
Needle filament protein	sctF	pscF	Q02KL0_PSEAB	85	AOZ12702	84	69.412	100	4.55E-38
PscF chaperone protein		pscG	Q02KL1_PSEAB	115	AOZ15082	119	58.407	94	2.05E-40
Type III secretion protein		pscH	Q02KL2_PSEAB	143	AOZ12703	137	42.857	89	2.40E-10
Inner rod protein	sctI	pscI	Q02KL3_PSEAB	113	AOZ12704	112	71.681	100	4.22E-55
Inner MS ring protein	sctJ	pscJ	Q02KL4_PSEAB	248	prot_1679	237	87.437	83	7.45E-131
Accessory cytosolic protein	sctK	pscK	Q02KL5_PSEAB	206	AOZ12705	208	54.902	98	2.18E-60
Stator	sctL	pscL	Q02KL6_PSEAB	231	AOZ12706	205	66.832	98	5.80E-101
Effector		exoU	Q02IF1_PSEAB	687	QPD07247.1	681	61.919	97	<1E-200
Chaperone		spcU	Q02IF2_PSEAB	137	AOZ12707	136	55.085	86	3.07E-42

a Unified nomenclature for conserved proteins of the type III secretion apparatus. Sct, secretion and cellular translocation (20, 21).

b Type III secretion system genes in Pseudomonas.

Our next step was to check if this T3SS gene locus is conserved across various strains of this bacterium or whether it is unique to clinical isolates or only this strain. To do this, we screened the genomes of several strains of P. lundensis, which were isolated from spoiled food sources (M101 and M105 from spoiled pasteurized milk in the United States; DSM6252 and MFPA15A12 from spoiled meat in Europe), cold environments (2T.2.5.2 from Antarctic temporary meltwater pond), and cystic fibrosis (AU10414 and AU11222). Strains AU1044, 2T.2.5.2, M101, and M105 are closed genomes that are annotated by the NCBI annotation pipeline, while the rest are present as draft genomes. Using the Psc family T3SS from PA14 as well as the newly identified T3SS genes from AU1044 as the reference, the 8 strains of P. lundensis were screened for similar genes, and BLAST results showed that all the strains of P. lundensis had a T3SS similar to strain AU1044. In all these clinical and environmental isolates, the identified T3SS genes were present as a single contiguous region and in the same order (Fig. 1C). Thus, we choose strain AU1044 as a representative strain of P. lundensis for the remainder of this study.

**FIG 2:**
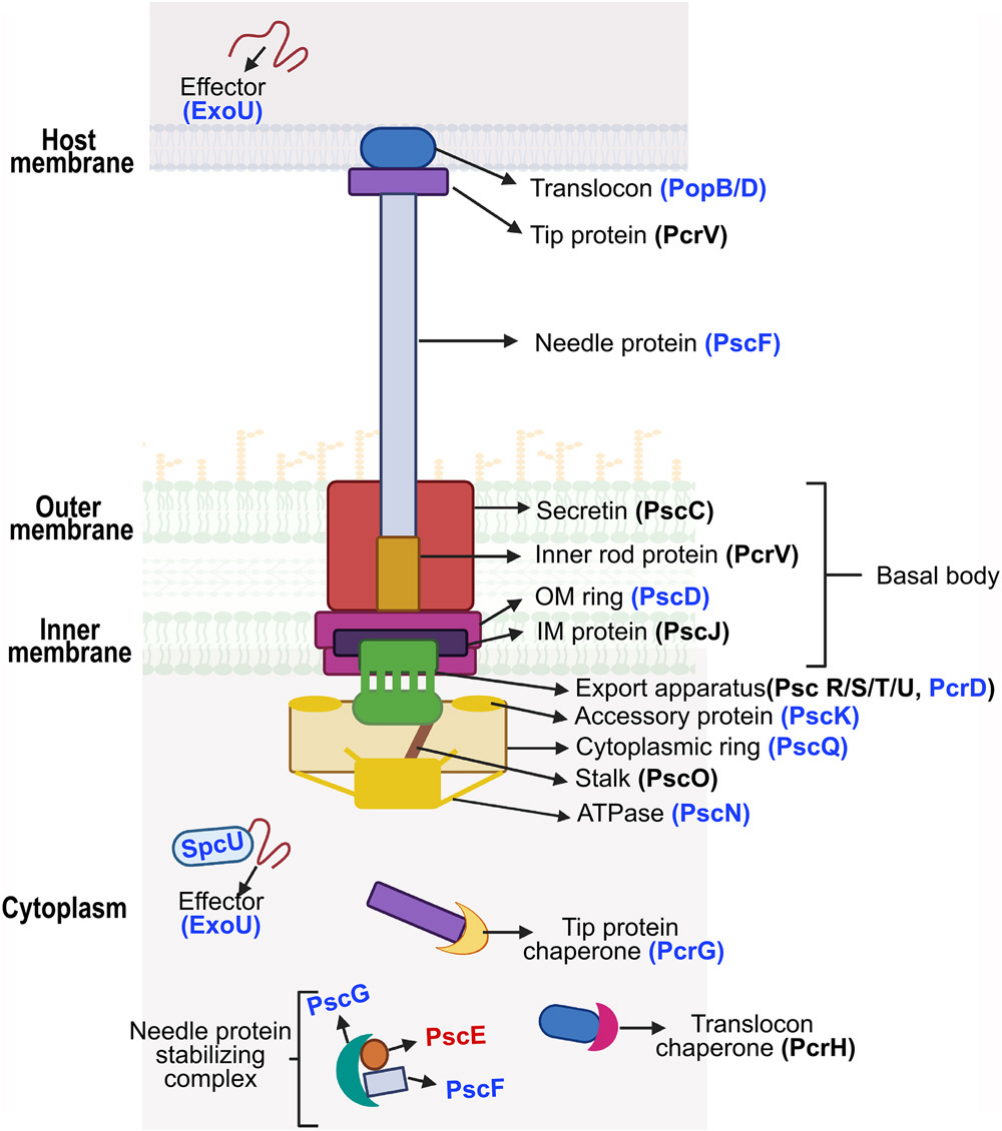
Schematic representation of the structural components of the type III secretion system in P. lundensis. The component labels are color coded depending on the protein alignment with their homologs in P. aeruginosa PA14. Black indicates an alignment score >75%, blue indicates alignment score <75% but >50%, and red indicates <50% alignment score. The schematics are based on the current knowledge of the complex and also presumed localizations for components that have so far not been unambiguously localized. IM, inner membrane; OM, outer membrane. Created with BioRender.com.

The genome of P. lundensis contains 38 genes belonging to Ysc/Psc family T3SS, clustered together as 5 consecutive operons and an effector-chaperone pair on the chromosome. *pscNOPQSTU*, *popNpcs1234DR*, *pcrGVH-popBD*, *excCEBA*, and *exsD-pscBCDEFGHIJKL* operons are the core T3SS genes that code for structural, secretory, and regulatory proteins, while the effector-chaperone pair (*exoUspcU*) encodes a type III effector protein (ExoU) and its respective chaperone (SpcU). Because P. lundensis falls under the same genus as P. aeruginosa, we used the same nomenclature for the T3SS genes found in P. lundensis as the Psc family T3SS in P. aeruginosa as well as the corresponding unified Sct (secretion and cellular translocation) designation (20, 21) (Fig. 2; Table 1). The organization of the core T3SS genes in P. lundensis is clustered together as five consecutive operons and shares some similarities with the Psc family T3SS in P. aeruginosa.

**FIG 3:**
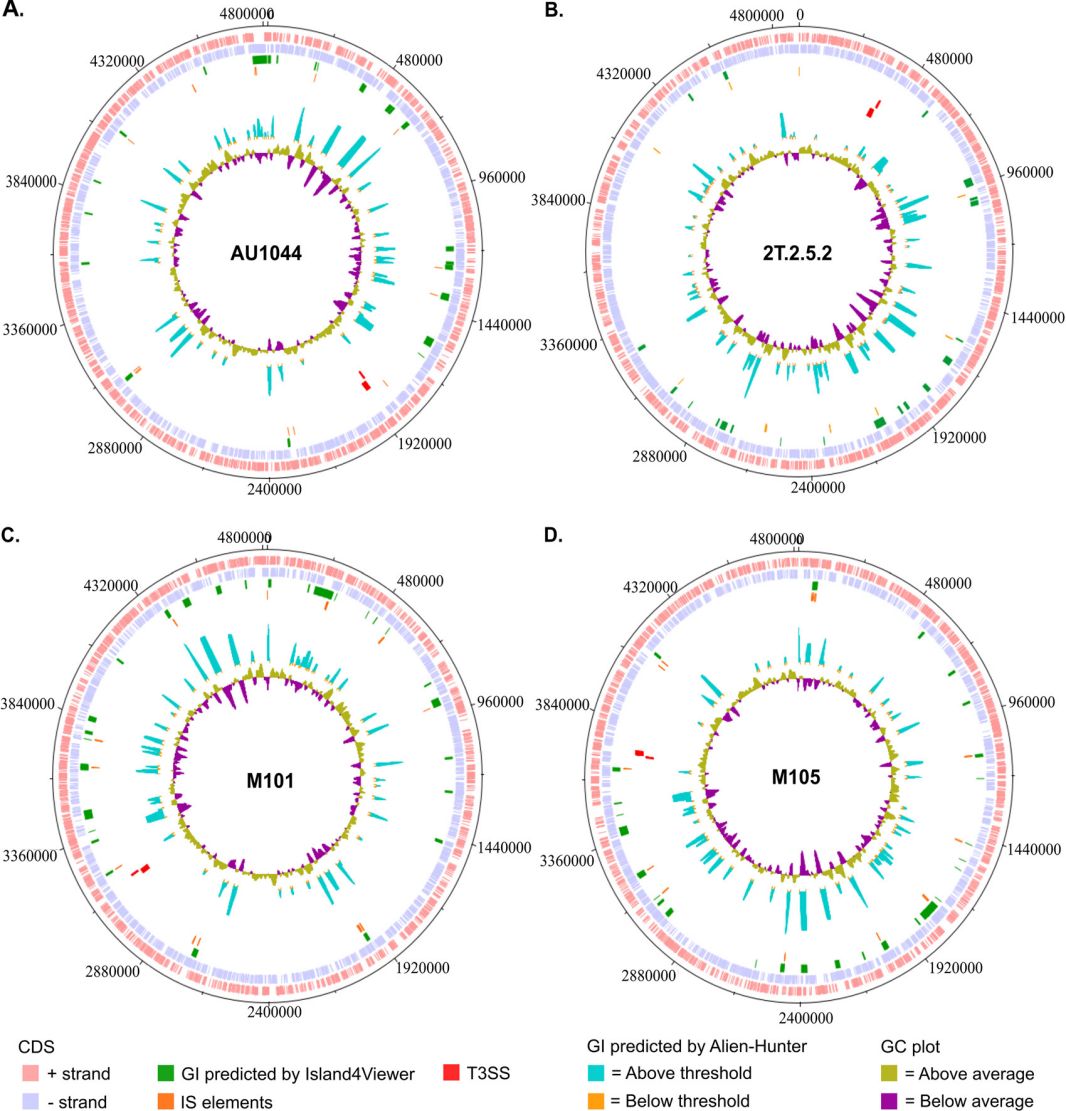
Circular genome plot of AU1044 (A), 2T.2.5.2 (B), M101 (C), and M105 (D). Counting the tracks from outside to inside, tracks 1 and 2 show the coding genes in the positive and negative strands, respectively; track 3 shows the genomic islands (GI), predicted using Island4Viewer; track 4 shows predicted insertion sequence (IS) elements predicted made using ISEScan; and gene blocks in red in tracks 5 and 6 are the type III secretion system genes in the positive and negative strands, respectively. Track 7, generated using Alien_Hunter (https://www.sanger.ac.uk/tool/alien-hunter/), shows GI that are predicted to be acquired through horizontal gene transfer (HGT). Blue peaks correspond to the probability of an HGT phenomenon. Track 8 is the GC plot; purple peaks are a segment of genomes with GC% below average, and olive-green peaks are segments with GC% above average. The T3SS genes do not lie in any predicted region of HGT or genomic islands.

However, there are some striking differences, the key one being the presence of the type III effector *exoU* immediately downstream to the structural genes. In species such as Yersinia pestis and Aeromonas hydrophilia where the T3SS is carried on the plasmid, the type III effectors and their chaperones are clustered together in the same plasmid, interspersed with other genes (22). However, when carried on the chromosome, type III effectors are generally found scattered. In P. aeruginosa, the 4 cytotoxic effectors are randomly distributed across the genome. In fact, in PA14, the ExoU effector and its chaperone SpcU are present in a pathogenicity island, PAPI-II (23). Screening AU1044 using a pathogenicity island prediction tool suggests that T3SS genes do not fall in pathogenicity islands (Fig. 3A). Screening other strains of P. lundensis (2T.2.5.2, M101, and M105) showed similar results (Fig. 3B to D) in that T3SS genes are not present in pathogenicity islands.

Another difference in T3SS gene organization in P. lundensis versus P. aeruginosa is the G+C content of this region. In P. aeruginosa, *the* G+C content of the T3SS is the same as that of the core genome (66.6%). In P. lundensis strains AU1044, 2T.2.5.2, M101. and M105, the G+C content of the T3SS is 61.75% ± 0.01% (standard deviation [SD]), which is different from the core genome of 58.67% ± 0.04% (SD). However, when using prediction tools (Alien_Hunter; https://www.sanger.ac.uk/tool/alien-hunter/), T3SS was not identified as a predicted region of horizontal gene transfer (HGT) (Fig. 3A). The distribution of insertion sequence (IS) elements in strain AU1044 also does not indicate any IS elements immediately upstream or downstream of the T3SS. A similar HGT prediction analysis was performed on the environmental and milk isolates of P. lundensis (Fig. 3B to D) and gave the same conclusion as for strain AU1044 in that T3SS genes do not appear in any predicted HGT regions.

### *In vitro* transcriptomics of the T3SS.

**FIG 4:**
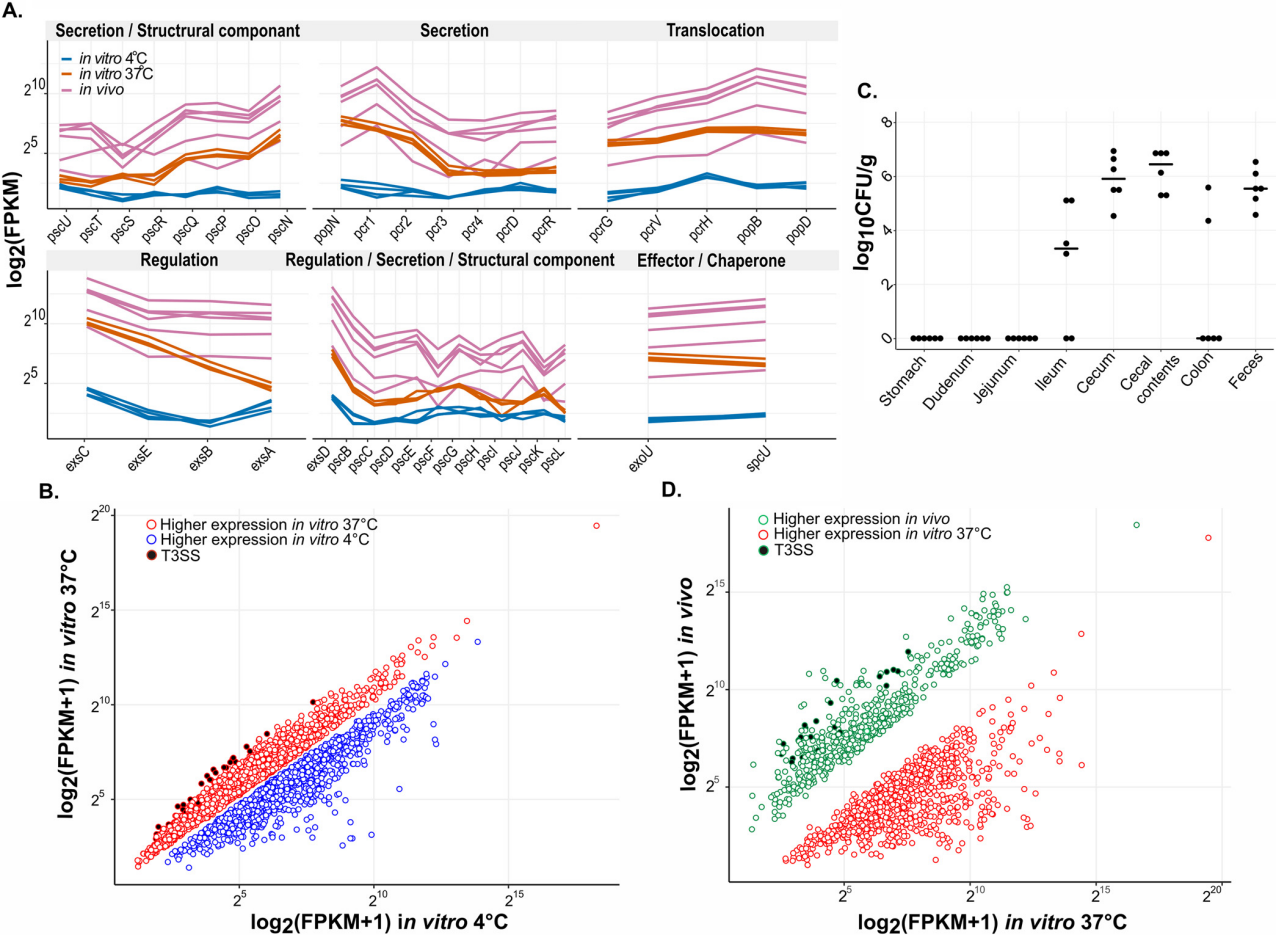
P. lundensis expresses its T3SS under both *in vitro* and *in vivo* conditions. (A) Expression profile of T3SS genes at 4°C and 37°C under *in vitro* growth conditions. The expression is shown in terms of FPKM reads that were normalized using DSEeq2. Here, we see an upregulation or increased FPKM levels of all T3SS genes at 37°C. Interestingly, some basal structure components, like PscU PscT, PscI, and PscL, have very small significant upregulation. Each line represents individual replicates/mice in either of the three conditions. (B) Scatterplot showing genes that have a significant change in expression at 37°C compared to 4°C. Open circles represent genes with a significant change in expression. Closed circles represent T3SS genes. (C) Levels of P. lundensis AU1044 in the intestinal tract of mice after oral gavage. Germfree BALB/c mice (*n* = 6) were given an oral gavage of 2.75 × 10^9^ CFU of AU1044 at day 0 and analyzed at 2 weeks postgavage. (D) Scatterplot showing genes that have a significant change in expression *in vivo* in the cecum of germfree mice compared to aerobic *in vitro* growth at 37°C. Genes were identified as differentially expressed if their adjusted *P* value (*Padj*) was less than 0.05 at a log_2_ fold change of >|0| for *the 37°C versus 4°C in vitro* experiment and >|1| for the *in vivo* versus 37°C *in vitro* experiment.

Our next objective was to investigate the transcriptional activity of the T3SS in P. lundensis. To do this, we set up an *in vitro* transcriptomics experiment. In P. aeruginosa, it is seen that temperature can be a T3SS-inducing condition where expression is seen at a human body temperature of 37°C but not at a lower temperature of 28°C (24). Hence, to investigate the transcriptional activity of T3SS in P. lundensis AU1044, the expression profiles of cells grown at different temperatures were compared. At the early stationary phase, P. lundensis AU1044 was able to actively transcribe several of its T3SS genes at 37°C and 21°C (Fig. 4A; data not shown; Table 2). When looking at the transcriptome of the P. lundensis in the two temperatures, at 37°C, the T3SS is among the top highly expressed genes compared to their expression at 4°C (Fig. 4B). On the other hand, the expression of several of these genes was almost undetectable at 4°C (Fig. 4A).

**TABLE 2:** Differential expression of T3SS genes in P. lundensis

Locus tag	Gene name	Gene expression data for:			
		37°C vs 4°C		*In vivo* vs *in vitro* 37°C	
		Log_2_ fold change	*P*_adj_ ^*c*^	Log_2_ fold change	*P*_adj_
AA042_08435	pscU^*a*^^,^^*b*^	1.00	6.21E-04	2.89	3.62E-03
AA042_08440	pscT^*a*^^,^^*b*^	1.47	4.17E-04	3.59	2.86E-05
AA042_08445	pscS^*a*^	4.36	8.98E-07	1.75	6.67E-01
AA042_08450	pscR^*a*^^,^^*b*^	2.32	5.00E-07	2.97	1.76E-02
AA042_08455	pscQ^*a*^	4.78	3.02E-47	2.41	3.19E-01
AA042_08460	pscP^*a*^	3.87	2.02E-27	1.94	7.39E-01
AA042_08465	pscO^*a*^	4.78	7.56E-27	2.19	9.99E-02
AA042_08470	pscN^*a*^	6.51	1.35E-94	1.88	7.62E-01
AA042_08475	popN^*a*^	6.02	4.88E-132	0.40	1.00E+00
AA042_08480	pcr1^*a*^	6.39	5.32E-19	2.73	1.72E-01
AA042_08485	pcr2^*a*^	5.53	2.90E-53	1.02	1.00E+00
AA042_08490	pcr3^*a*^	4.28	5.85E-12	2.39	1.75E-01
AA042_08495	pcr4^*a*^^,^^*b*^	2.33	7.99E-07	2.64	5.00E-02
AA042_08500	pcrD^*a*^^,^^*b*^	1.68	8.56E-11	3.05	2.09E-03
AA042_08505	pcrR^*a*^^,^^*b*^	2.69	1.34E-11	3.18	1.31E-03
AA042_08510	pcrG^*a*^	6.39	9.49E-15	0.70	1.00E+00
AA042_08515	pcrV^*a*^	5.05	5.57E-91	1.60	1.00E+00
AA042_08520	pcrH^*a*^	4.16	7.19E-89	1.22	1.00E+00
AA042_08525	popB^*a*^	5.36	2.24E-159	2.80	1.93E-01
AA042_08530	popD^*a*^	4.94	1.05E-88	2.29	4.59E-01
AA042_08535	exsC^*a*^	5.89	2.29E-163	1.70	5.27E-01
AA042_08540	exsE^*a*^	6.59	4.70E-96	1.52	1.00E+00
AA042_08545	exsB^*a*^^,^^*b*^	6.07	3.92E-52	3.31	2.25E-02
AA042_08550	exsA^*a*^^,^^*b*^	1.66	4.00E-08	4.99	8.43E-06
AA042_08555	exsD^*a*^^,^^*b*^	3.74	3.53E-88	3.30	3.78E-02
AA042_08560	pscB^*a*^^,^^*b*^	3.21	5.01E-16	3.61	1.53E-02
AA042_08565	pscC^*a*^^,^^*b*^	2.65	2.86E-24	3.37	2.37E-02
AA042_08570	pscD^*a*^^,^^*b*^	2.21	4.38E-17	3.58	1.27E-02
AA042_08575	pscE^*a*^^,^^*b*^	2.54	4.98E-07	3.62	2.87E-05
AA042_08580	pscF^*a*^	2.08	1.03E-08	0.95	1.00E+00
AA042_08585	pscG^*a*^	2.49	1.69E-18	2.07	2.13E-01
AA042_08590	pscH^*a*^^,^^*b*^	1.66	2.47E-05	2.60	2.32E-02
AA042_08595	pscI^*a*^^,^^*b*^	1.32	7.35E-03	3.48	3.46E-05
AA042_08600	pscJ^*a*^^,^^*b*^	1.34	1.49E-05	4.11	5.55E-08
AA042_08605	pscK^*a*^	2.16	7.36E-17	0.74	1.00E+00
AA042_08610	pscL^*a*^^,^^*b*^	0.92	2.49E-02	3.93	7.70E-05
AA042_08615	exoU^*a*^	4.94	8.66E-74	2.60	3.23E-01
AA042_08620	spcU^*a*^	6.32	2.66E-169	1.40	1.00E+00

a Gene with significant change in expression at 37°C versus 4°C.

b Gene with significant change in expression at *in vivo* versus *in vitro* 37°C.

c *P*_adj_, adjusted *P* value, generated though DESeq2 differential gene expression analysis.

There is a significant upregulation of T3SS genes at 37°C compared to 4°C (Fig. 4A). Some T3SS genes have >5-log_2_ fold changes in expression, for example, the T3SS assembly regulatory gene *exsB* (25), ATPase *pscN* (26), chaperone and cytoplasmic regulator *pcrG* (27), effector *exoU*, T3SS negative regulator *exsE* (28), and negative regulators of exoenzyme secretion *popN* and *pcr2* (29). A few genes have a <1.5-log_2_ fold change. These include ATPase regulator *pscL* (30), basal body component *pscU*, basal body rod component *pscI* (31), basal structure component *pscJ* (26), membrane component of the export apparatus *pscT* (32), secretion protein *pscH*, and inner membrane component *pcrD* (21). Different levels of upregulation were also seen in the expression of translocation proteins *popB* and *popD*, tip protein *pcrV* (33), and the needle protein *pscF* (34), all components of the injectosome necessary for exporting effectors into host cells. Thus, the *in vitro* transcriptomics data demonstrated that the T3SS in P. lundensis is transcriptionally active at 37°C.

### *In vivo* transcriptomics of the T3SS.

Our next objective was to study whether the host environment has an effect on the expression profile of T3SS at 37°C. Since P. lundensis is a common milk contaminant, the most common route of exposure to this bacterium would be through the consumption of milk. Therefore, we decided to study the expression profile of P. lundensis in the intestinal tract (by employing monocolonization of germfree mice) and compare it to the expression of bacterial cells cultured *in vitro*. P. lundensis was able to successfully colonize the gut of all the germfree mice after a single oral gavage (Fig. 4C) without any histological or clinical signs of disease (data not shown). The cecum and feces, collected after 14 days of inoculation, had an average level of 10^5^ CFU/g. Cecal content (CC) collected from the lumen of the cecum had the highest level of colonization, with an average of 10^6^ CFU/g. The ileum had low-level colonization, while the remaining two samples had undetectable levels. Interestingly, no colonization was seen in the stomach, duodenum, and jejunum. We used the cecal contents of germfree mice colonized for 14 days with strain AU1044 as the samples for transcriptomics analysis. Compared to the expression profile seen *in vitro* at 37°C, several genes had higher expression *in vivo*, including T3SS genes (Fig. 4A and D). Several T3SS genes had either increased expression levels or no significant change in expression levels *in vivo* compared to their expression *in vitro* at 37°C. Some of the genes having higher expression *in vivo* include genes coding for export apparatus *pscT*, *pscU*, and *pcrD*; basal body components *pscJ*, *pscI*, and *pscD*; and outer membrane ring protein *pscC*. *exs*A also had significant upregulation of 4.9-log_2_ fold change (Fig. 4A; Table 2). Other regulatory genes, such as *exsD* and *exsB*, also had greater than 3-log_2_ fold change in expression. These data clearly indicate that P. lundensis can express its T3SS when colonizing a mammalian host.

## DISCUSSION

In this paper, we have demonstrated the presence of a transcriptionally active Ysc family T3SS in a non-*aeruginosa* Pseudomonas species (NAPS), P. lundensis. Isolates from clinical samples and various food spoilage sources, as well as an environmental isolate from an Antarctic temporary meltwater pond, carry the Ysc family T3SS. P. lundensis also encodes an ExoU type III effector protein and its respective chaperone (SpcU), and genes for these proteins are present immediately downstream to the core T3SS genes. Our transcriptomics analysis demonstrates that under *in vitro* aerobic growth conditions, the T3SS in the clinical isolate AU1044 is expressed at 37°C, while the expression of the system is negligible at 4°C. When in the cecal lumen of monocolonized germfree mice, P. lundensis also transcribes its T3SS, and several genes were more highly expressed than at 37°C *in vitro*.

Most species in the Pseudomonas fluorescens species complex have an Hrp1 family T3SS. However, apart from the Hrp family, root-associated strains of P. fluorescens also carry an Rsp family T3SS (11), and P. fluorescens strain K113 has both the Hrp1 and SPI-1 family (Salmonella pathogenicity island) T3SS (35). Though P. aeruginosa is the only Pseudomonas species to have a well-characterized Ysc family T3SS, there have been previous reports of P. fluorescens complex species carrying individual genes that are highly homologues to those within the Ysc family; P. baetica has core Ysc family T3SS gene homologs (36); P. weihenstephanensis has ExoU homologs (37). A phylogenetic tree based on multilocus sequence analysis shows that the P. fluorescens group is distant from P. aeruginosa (7, 38). Though the functionality of the T3SS in these NAPS is not known, it raises interesting questions about the evolution of T3SS among the Pseudomonas species.

Among the strains studied here, out of the four known cytotoxic type III effectors in P. aeruginosa, P. lundensis only carries a homologue of the *exoU* gene. The T3SS genes found in P. lundensis all have high sequence similarity with those in P. aeruginosa. The occurrence of all four cytotoxic effector proteins in a single strain of P. aeruginosa strain is very rare. Most clinical isolates of P. aeruginosa code for either ExoU/ExoT or ExoS/ExoT. Out of the 4 effectors (ExoU, ExoS, ExoT, and ExoY), ExoU has the greatest impact on disease severity in humans (39). ExoU is a potent cytotoxin with phospholipase A_2_ activity (16, 17, 39). Although only an *exoU* homologue was identified in P. lundensis, we also looked for genes with an upstream ExsA-activated promoter because all genes of the T3SS are regulated by the ExsA promoter, and the promoter-binding sequence is highly conserved across Ysc family T3SS (40). Preliminary analysis of scanning the genome for the conserved ExsA-binding motif (41) identified four putative type III effectors (DNA polymerase IV, TonB-dependent receptor, heme-binding protein, and response regulator transcription factor) (data not shown). However, it is important to note that ExsA has also been reported to regulate the expression of non-T3SS genes, including the metalloprotease *impA* and other secretory proteins in P. aeruginosa (42). Thus, it remains to be determined biochemically if there are additional T3SS effector proteins in P. lundensis, but there are some potential targets.

P. lundensis grown aerobically at 4°C in LB broth was unable to express several T3SS genes. P. lundensis is primarily a psychrotrophic bacterium, and T3SS is conserved in all the strains studied here. Our *in vitro* growth set up at 4°C may not be an accurate representation of the various psychrotrophic niches that this bacterium inhabits. Though T3SS is a well-known virulence factor in P. aeruginosa, there are reports which show that P. aeruginosa uses T3SS to colonize and evade predation by protozoa by killing biofilm-associated amoebae. (18). Though it is unclear what role T3SS may play in the various niches that P. lundensis colonizes, it is intriguing to postulate that in P. lundensis, the T3SS may play a similar role in protecting the bacterium from environmental phagocytes.

At human body temperature, P. lundensis expresses its T3SS. The translocation pore of the T3SS needle complex, PopB/PopD, which can cause host cell injury independent of the effectors (43, 44) as well as the cytotoxic effector, ExoU, is expressed at human body temperature. However, the various levels of expression seen across the system suggest that not all T3SS genes are transcribed at the same rate. The T3SS global transcription regulator *exsA* has a 1.6-log_2_ fold change increase in expression at 37°C compared to growth at 4°C. The activity of ExsA protein, the global regulator of T3SS, is controlled by levels of anti-activator ExsD, anti-anti-activator ExsC, and secreted protein ExsE (41, 45). Upon induction of T3SS, ExsE gets secreted, thereby decreasing the cytosolic levels of the protein. This leads to increased affinity of ExsC and ExsD, resulting in the release of ExsA from ExsD. Our data show that the upregulation of e*xsA* at 37°C is less than the genes involved in its regulation (*exsD*, *exsC*, and *exsE*). The PcrG protein acts as both a chaperone for the tip protein PcrV as well as a negative regulator of the effector export. PcrG forms a complex with PcrN, Pcr1, and chaperon protein Pcr2, and this complex docks of PcrD, effectively blocking the export of effectors (27). At 37°C, the negative regulators of the system, *pcrG*, *pcrV*, *pcr1*, and *pcr2*, are among the highly expressed genes. Interestingly, a similar expression pattern of the T3SS regulatory genes is reported in Vibrio parahaemolyticus (46). In both, upon increased growth temperature for P. lundensis and increased cytotoxic activity in V. parahaemolyticus, upregulation in the expression of *exsA* is relatively low compared to *exsD*, *exsC*, and *exsE.* Similarly, in both instances, there is an upregulation of genes that are known to block the export of effectors.

While direct studies remain to be performed with P. lundensis, the studies in V. parahaemolyticus provide support for the hypothesis that the T3SS of P. lundensis confers cytotoxic activity.

From our transcriptomics data, we see that the T3SS is expressed at high levels both during *in vivo* and *in vitro* growth conditions at 37°C but not at 4°C *in vitro*. The T3SS is expressed *in vitro* at 21°C at levels slightly lower than at 37°C but much higher than at 4°C (data not shown), so it remains to be determined whether there is a temperature “switch” for the upregulation of the T3SS or whether it generally increases between 4°C and 37°C. P. lundensis AU1044 isolated from the sputum samples of CF patients can successfully colonize and grow in the anaerobic environment of the gut of germfree mice as well as aerobically *in vitro* in LB broth. These niches present various environmental pressures to the bacterium like micro- and macronutrients, pH, and oxygen conditions as well as carbon sources, all suggesting that P. lundensis AU1044 isolates exhibit metabolic diversity. Comparative analysis of P. lundensis strains shows that CF isolate AU1044 shares 99% sequence similarity with strains isolated from food samples as well as Antarctic temporary meltwater ponds. In other preliminary studies, we have also been able to identify 16S rRNA gene sequences that include P. lundensis in data sets from patients with chronic obstructive pulmonary disease and interstitial pulmonary fibrosis but not in healthy human lungs. As a potent hydrocarbon degrader, the ability of P. lundensis to degrade aromatic hydrocarbons (47) could facilitate its colonization of the lungs because pulmonary surfactant is largely composed of phospholipid. Despite the expression of a T3SS *in vivo*, it was intriguing that germfree mice inoculated with P. lundensis did not show any histological evidence of inflammation or disease in the ileum, cecum, or colon after 14 days of colonization (*n* = 6; histological score, 0.0 ± 0.0; 0 to 12 scale as described in Materials and Methods). We did not investigate whether there are changes in host mucosal gene expression in these mice. Thus, further investigation is merited on the nature of the symbiotic interaction of P. lundensis with a mammalian host. There is one report of the oral administration of P. lundensis to healthy humans (as a negative control in a probiotic bacteria study) (48). In this study, the subjects did not have ill effects on their health after 2 weeks of oral administration of P. lundensis (at a concentration proposed to mimic the levels in 1,000 mL of milk). With the identification of a complete Ysc T3SS in P. lundensis that is expressed at 37°C *in vivo*, we wonder whether this bacterium has some level of interaction with the gastrointestinal tract after consumption by a mammalian host. It remains to be determined what environmental/evolutionary pressures act to retain the T3SS in P. lundensis during growth in the environment since mammalian hosts are not a primary environment for this bacterium.

## MATERIALS AND METHODS

### Bacterial genomic sequences used for analysis.

Bacterial genomic sequences used include Pseudomonas aeruginosa UCBPP-PA14 (Assembly accession no. GCF_000014625.1) (reference strain), Pseudomonas lundensis AU1044 (GenBank accession no. CP017687) (reference strain), 2T.2.5.2 (GenBank accession no. NZ_CP062158.2), M101 (GenBank accession no. CP075177), M105 (GenBank accession no. CP075180), DSM6252 (GenBank accession no. NZ_JYKY00000000), AU11122 (GenBank accession no. LCYV00000000), AU10414 (GenBank accession no. LCYU00000000), and MFPA15A1205 (GenBank accession NZ_OBKZ00000000). AU1044 is deposited as DSM 103277 in the DSMZ culture collection in Germany.

### *In vitro* growth.

**FIG 5:**
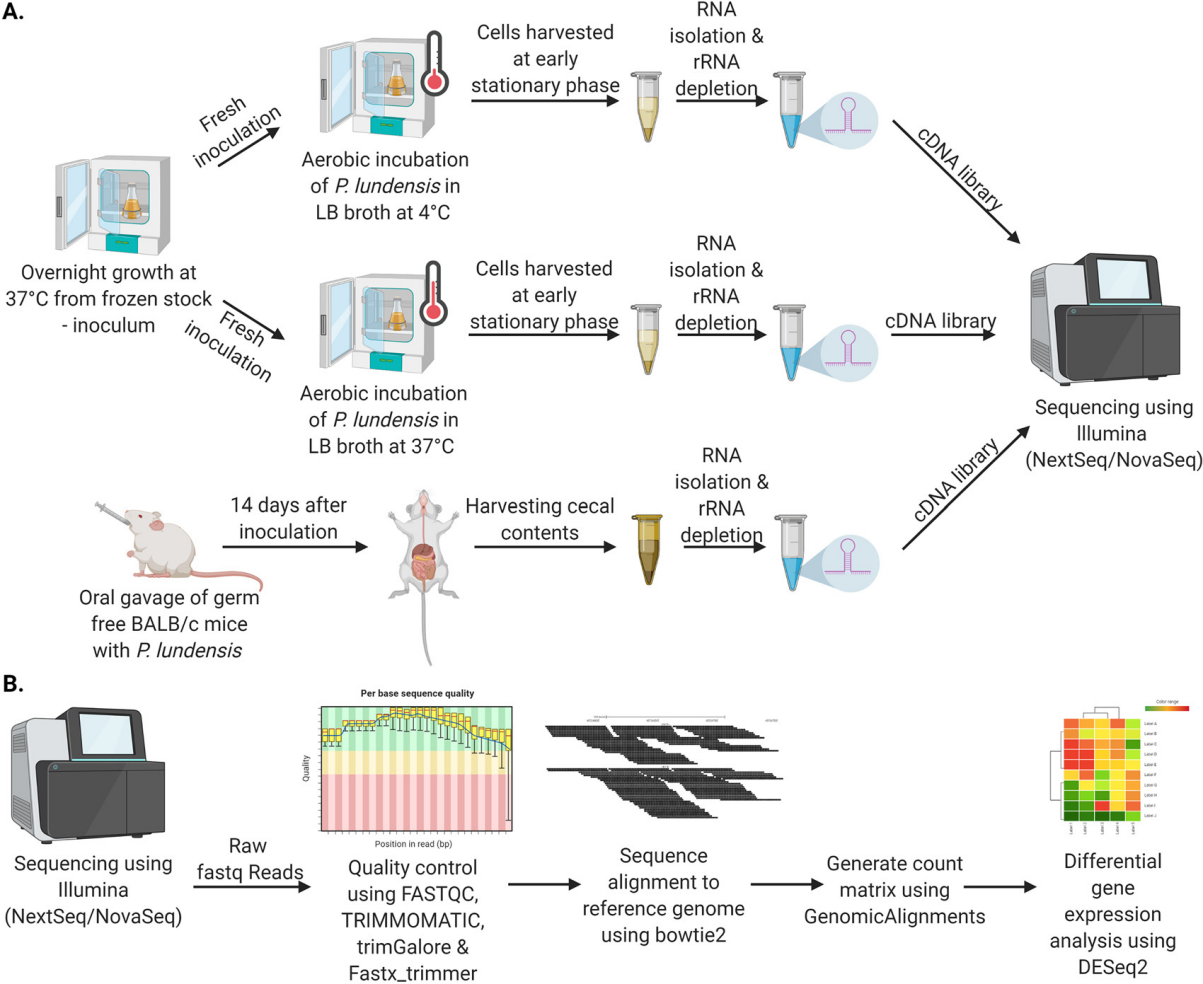
Schematic representation showing the outline for the experiments set up for *in vitro* and *in vivo* RNA transcriptomics (A) and the bioinformatics pipeline used to analyze the data (B).

The experiment was set up as shown in Fig. 5A using the AU1044 strain. Frozen overnight culture of AU1044 from frozen stock was set up in 20 mL of Luria Bertani (LB) broth (Thermo Fisher) at 37°C with shaking at 120 rpm. Twenty microliters of this culture were used to inoculate triplicates of 20 mL LB broth, incubated at 4°C, and 37°C with shaking at 120 rpm. Bacterial cells (average cell count of 5 × 10^6^) were harvested once the culture reached an optical density (OD) of 2.1 at 600 nm. Cells were treated with Qiagen’s RNAprotect bacteria reagent at a 2:1 ratio of RNAprotect for every volume of sample and then stored at −80°C until RNA isolation.

### Gnotobiotic animal colonization experiment.

The experiment was set up as shown in Fig. 5A. All germfree mouse experiments were handled at the germfree animal facility at the University of Michigan. Ten- to 12-week-old, female germfree BALB/c mice underwent gastric colonization through oral gavage of 2.75 × 10^9^ CFU/mL of P. lundensis AU1044 (*n* = 6). At 14 days postcolonization, the stomach, small intestine proximal and distal ends, cecal content, cecum, colon, and feces were collected for CFU plating and cecal contents for RNA isolation.

### RNA extraction and RNA sequencing.

*In vitro* samples from −80 were first thawed and then incubated with 200 μL of lysozyme (concentration of 1 mg/mL of Tris-EDTA [TE] buffer) for 10 min in a shaker. After cell lysis, RNA isolation was done using Qiagen’s RNeasy minikit. RNA isolation from the cecal contents of colonized mice was performed using phenol-chloroform extraction followed by clean-up using RNeasy minikit as previously described (49). Isolated RNA samples were given for sequencing at the DNA sequencing core at the University of Michigan. Ribosomal depletion and library preparation were done by the core using the NEBNext depletion kit and the NEBNext Ultra II RNA library prep kit, respectively. *In vitro* samples were sequenced using Illumina NextSeq 500 and *in vivo* samples using NovaSeq 6000 both at 300 cycles with mid-sequencing output.

### RNA sequencing data analysis.

The schematic representation of the analysis pipeline is shown in Fig. 5B. Raw RNA sequencing reads were quality trimmed using Trimmomatic 0.39 with LEADING and TRAILING set at 20 and MINLEN set at 100. TrimGalore (version 0.6.5) and Fastx_trimmer (version 0.0.14) tools were used to remove and trim out adapter reads from the 3′ end, respectively. The reads were aligned to the P. lundensis strain AU1044 using Bowtie2 version 2.3.4.3 with default parameters for an end-to-end alignment. The resulting SAM files were converted to BAM files using SAMtools version 1.7. Count matrix was generated in Rstudio using the GenomicAlignments package. Fragments per kilobase of transcript per million mapped reads (FPKM) reads were generated, and differentially expressed genes were identified both using DESeq2. Genes were identified as differentially expressed if their adjusted *P* value (*P*_adj_) was less than 0.05 at a log_2_ fold change of >|0| for the 37°C versus 4°C *in vitro* experiment and >|1| for the *in vivo* versus 37°C *in vitro* experiment.

### Genomic analysis.

GC island prediction and pathogenicity island prediction were done using Alien_Hunter (50) and IslandViewer4 (51) at default parameters. Scanning for insertion sequence elements was done using ISEScan (52). The T3SS genes in AU1044 were identified using BLAST with Pseudomonas aeruginosa UCBPP-PA14 strain as the reference. For generating the gene maps of T3SSs in different strains of P. lundensis, the GFF files from NCBI and RAST annotations were downloaded for completely assembly and draft genomes, respectively. The maps were generated in RStudio using genoPlotR (53). The direction of the T3SS operons differs between the strains; however, we speculate that this might be due to a difference in annotations as the difference in the positive- and negative-strand genes extend to the genes upstream and downstream to the T3SS for P. lundensis strains.

### Histology and histological scoring.

Intestinal tissue was fixed in 10% formalin for at least 24 h and then transferred to 70% ethanol. Tissue was processed, paraffin embedded, sectioned, and used to prepare hematoxylin and eosin (H&E)-stained slides. All section were analyzed at ×4, ×20, and ×400 magnifications. Light microscopic evaluation of H&E-stained ileum, cecum, and colonic sections was performed using a previously established system (54). Slides were scored (0 [none] to 4 [extensive]) for mononuclear and neutrophilic inflammation, edema, and epithelial damage as defined below.

### (i) Inflammation.

For inflammation, 0 represents no inflammation; 1 represents minimal, multifocal mononuclear, or neutrophilic infiltration; 2 represents moderate, multifocal mononuclear, or neutrophilic infiltration (greater submucosal involvement); 3 represents severe multifocal to coalescing mononuclear or neutrophilic infiltration (greater submucosal with or without mural involvement); and 4 is the same as 3 but with abscesses or extensive transmural involvement.

### (ii) Edema.

For edema, 0 represents no edema; 1 represents mild, focal, or multifocal edema with minimal submucosal expansion (<2×); 2 represents moderate multifocal edema with moderate submucosal expansion (2 to 3×); 3 represents severe multifocal to coalescing edema with severe submucosal expansion (>3×); and 4 is the same as 3 but with diffuse submucosal expansion.

### (iii) Epithelial damage.

For epithelial damage, 0 represents no epithelial damage; 1 represents mild multifocal, superficial damage (vacuolation, increased apoptosis, villus tip attenuation/necrosis); 2 represents moderate, multifocal superficial damage (same qualitative changes as above); 3 represents severe multifocal to coalescing mucosal damage with or without intraluminal aggregate of inflammatory cells and sloughed epithelium or ulcerated mucosa); and 4 is the same as 3 but with extensive epithelial destruction or ulcer formation.

### Data availability.

*In vitro* and *in vivo* RNA sequencing (RNA-seq) data reported in this paper have been deposited in NCBI’s GEO repository under the BioProject identifier PRJNA733127 (accession numbers SAMN19367880 to SAMN19367893).
